# Clinimetrics of the Upright Motor Control Test in chronic stroke

**DOI:** 10.1002/brb3.826

**Published:** 2017-09-08

**Authors:** Frances Rom M. Lunar, Edward James R. Gorgon, Rolando T. Lazaro

**Affiliations:** ^1^ Department of Physical Therapy University of the Philippines Manila Manila Philippines; ^2^ Department of Physical Therapy Samuel Merritt University Oakland CA USA; ^3^ Department of Physical Therapy California State University Sacramento Sacramento CA USA

**Keywords:** movement functions, muscle function, reliability, reproducibility of results, stroke rehabilitation

## Abstract

**Introduction:**

Insufficient literature exists regarding the clinimetric properties of the Upright Motor Control Test Knee Extension and Flexion subtests (UMCT‐KE and UMCT‐KF, respectively). This study examined the interrater and test‐retest reliability of these subtests, and determined the relationship between the UMCT‐KE and a clinical measure of muscle function in a sample of adults with chronic stroke.

**Methods:**

Three raters independently administered the UMCT‐KE and UMCT‐KF on adults with chronic stroke with spasticity/abnormal movement patterns. Testing of each participant occurred on two occasions (T1 and T2) separated by a two‐week interval. A fourth rater independently administered the Five Times Sit to Stand Test (FTSST), a measure of lower extremity muscle function (power), on T2.

**Results:**

Twenty‐nine adults aged 55 ± 8 years, comprising 21 men (72%), and who were 9 ± 5 years poststroke, completed the study. Most of the participants (66%, 19/29) did not require an assistive device during walking. The UMCT‐KE and UMCT‐KF demonstrated substantial interrater reliability (*W *=* *0.63–0.67 and 0.72–0.75, respectively) and substantial to almost perfect test‐retest reliability across the raters (*W *=* *0.75–0.82 and 0.85–0.87, respectively). The UMCT‐KE showed positive inverse correlation with the FTSST (ρ = −0.52, *p *=* *.003).

**Conclusions:**

Scores on both subtests are reproducible within raters and across different raters. The relationship of UMCT‐KE scores with FTSST scores implies that the UMCT‐KE can provide information that relates with the construct of muscle function in a weight‐bearing position.

## 1 INTRODUCTION

Voluntary control of the lower limbs is an important factor for controlling the upright position (Arya, Pandian, Abhilasha, & Verma, 2014). Impairment in voluntary control of movement exists among individuals with stroke and persists well beyond the inpatient rehabilitation period (Neckel, Pelliccio, Nichols, & Hidler, 2006). The Upright Motor Control Test (UMCT) (Hislop & Montgomery, 2007) or Upright Control Test (Keenan, Perry, & Jordan, 1984) was originally developed at the Rancho Los Amigos Hospital in Downey, California to assess voluntary control of the paretic lower limb. The test was first described in 1983 in an unpublished manuscript by Toman (Toman, unpublished thesis). It has been used to determine the influence of muscle tone on lower extremity control in an upright position in individuals with neurologic injury (Barto, Gronley, Perry, & Yoshida, 1985; Keenan et al., 1984; Perry et al., 1995; Sweeney & Smutok, 1983). It has also been used as an outcome measure in stroke clinical trials as a measure of functional muscle strength (Almarez, Macatangay, Santos, & Flores, 2010; Ambrosini, Ferrante, Pedrocchi, Ferrigno, & Molteni, 2011; Ferrante, Pedrocchi, Ferrigno, & Molteni, 2008). A recent systematic review has reported the practical advantages of using the UMCT, such as short administration time, simple instrumentation, and uncomplicated testing procedures (Gorgon & Lazaro, 2016). Therefore, the UMCT can be used in a range of practice settings especially those with limited resources (Gorgon & Lazaro, 2016).

Among the subtests of the UMCT, the Knee Extension (UMCT‐KE), and Flexion (UMCT‐KF) subtests have been suggested to be reflective of total voluntary lower extremity control and not just voluntary control of the knee joint muscles (Perry et al., 1995). The UMCT‐KE provides information regarding supportive functions of the leg during the stance phase of walking (Ade, Smith, & Spigel, 2012). The UMCT‐KF provides information regarding control of leg advancement during the swing phase of walking (Ade et al., 2012). However, no reliability studies have been published to support the reproducibility of test results (Gorgon & Lazaro, 2016). Inability to determine the level of reproducibility of test results will adversely affect the usability of a test and, ultimately, the quality of assessment and treatment.

Both the UMCT‐KE and UMCT‐KF measure voluntary control of movement. In the International Classification of Functioning, Disability and Health (ICF), control of voluntary movement is a body function classified under the domain of Neuromusculoskeletal and Movement‐related Functions (World Health Organization, 2001). Within the Neuromusculoskeletal and Movement‐related Functions domain, it is specifically covered under the Movement Functions category. To further determine the value of the UMCT, its relationship with other concepts within the same ICF domain needs to be investigated. Scores on the UMCT have been found to correlate with peak vertical ground reaction forces, a laboratory measure of supportive functions of the leg (Mercer et al., 2009). However, how it relates with other categories within the same ICF domain, such as muscle functions, has not been studied. According to the ICF, muscle functions include force generated by contraction of a muscle/muscle group (World Health Organization, 2001) and impaired muscle function has been linked to difficulty in movement control in people with stroke (Neckel et al., 2006). Knowledge of such relationships can guide clinicians in using information from the UMCT in determining outcomes and designing interventions.

Thus, this study aimed to: (1) determine the interrater and test‐retest reliability of the UMCT‐KE and UMCT‐KF, and (2) determine the relationship of the UMCT‐KE with a clinical measure of muscle function.

## 2 MATERIALS AND METHODS

This study used an observational methodological design (Portney & Watkins, 2009). The University of Philippines Manila Research Ethics Board approved the study protocol. All participants provided written informed consent.

Adults with chronic stroke were conveniently sampled from a multidisciplinary outpatient therapy clinic and stroke support group in an urban area in the Philippines. The inclusion criteria were: (1) age of at least 18 years; (2) presence of chronic stroke (6 months or more had elapsed since stroke diagnosis); (3) ability to follow at least three unrelated commands; (4) ability to maintain standing with or without physical assistance; and (5) presence of lower extremity spasticity (Modified Modified Ashworth Scale score of 1 or higher (Abolhasani et al., 2012)) or abnormal movement synergy (inability to isolate lower extremity joint movements on command). Potential participants were excluded if they: (1) were medically diagnosed with severe visual, hearing, cognitive, behavioral, or receptive language impairment; (2) had major sensory deficits; (3) had a recent lower extremity musculoskeletal injury; or (4) had bilateral stroke.

**Figure 1:**
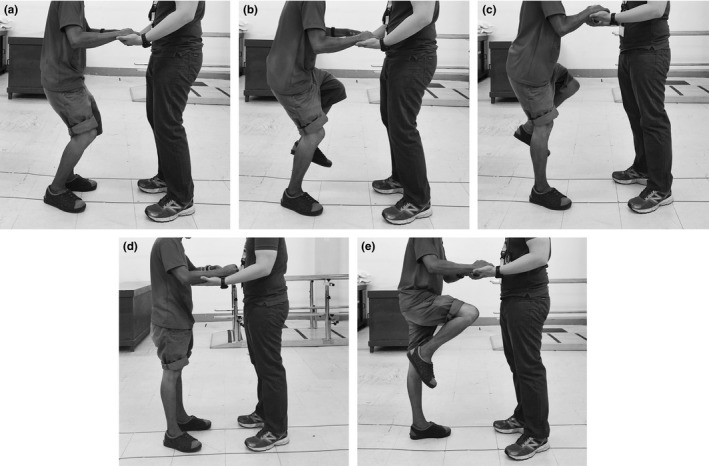
A patient with right‐sided hemiparesis performing the UMCT‐KE (a–c) and UMCT‐KF (d–e)

The UMCT‐KE and UMCT‐KF were administered following the procedure described by Perry et al. (Figure 1) (Perry et al., 1995). For the UMCT‐KE, the patient stood with knees in 30 degrees of flexion. The patient then transferred full weight on the more affected lower extremity by lifting the opposite foot off the floor. Finally, the patient attempted to extend the more affected knee. The rater scored knee extensor strength using a three‐point ordinal scale: 3 or “strong,” (can support the body weight by fully extending the knee from flexion); 2 or “moderate,” (can support the body weight on the flexed knee without collapsing into further flexion but cannot control extension); and 1 or “weak,” (cannot support the body weight on the flexed knee or the test knee collapses into further flexion). For the UMCT‐KF, the patient stood with knees in extension. The patient then brought the knee of the more affected lower extremity up toward the chest as high and fast as possible three times. The rater scored knee flexor strength using a two‐point ordinal scale: 3 or “strong,” (can flex the knee to >60 degrees, three times in 10 s); and 1 or “weak,” (cannot flex the knee to >60 degrees, three times in 10 s, or cannot move the lower limb at all). If required for either procedure, assistance was given in maintaining balance by allowing the participant to place the palm or forearm on the rater's palm or forearm. However, if a participant needed to bear excessive weight on the rater to complete the task, a lower score was given. Verbal cues were provided if necessary but were generally avoided.

Scores on the UMCT‐KE were related to scores on the Five Times Sit to Stand Test (FTSST), a commonly used clinical test of muscle function. The test involved recording the time it took for a participant to complete five repetitions of the sit‐to‐stand task, with a shorter time reflecting better performance (Mong, Teo, & Ng, 2010). Participants were encouraged to not use their hands to help push up during the test. Although it has been regarded as a reliable and valid measure of functional lower extremity strength in adults with chronic stroke (Mong et al., 2010), the FTSST involves force production of muscles used in sit‐to‐stand within a given time constraint and is therefore a measure of muscle power. As the sit‐to‐stand maneuver involves use of the lower extremity muscles, most notably the knee extensor group (Bohannon, Bubela, Magasi, Wang, & Gershon, 2010), in a weight‐bearing position, FTSST scores were tested for correlation with UMCT‐KE scores.

Raters obtained data on the UMCT‐KE and UMCT‐KF on two testing sessions (T1 and T2) that were separated by a two‐week time interval. Three physical therapists of varying years of clinical experience administered the tests. Prior to testing, all raters underwent training on test administration and scoring. Testing sessions were conducted in a well‐lit, cool, and quiet room in an outpatient therapy clinic. The order of raters was counterbalanced. Similar testing conditions were observed on all occasions. On T2, raters were blinded to the UMCT scores of the participants from T1. A fourth physical therapist who was blinded to the UMCT scores of the participants administered the FTSST on T2. For all tests, participants were given one practice trial, followed by two test trials. Scores were recorded and best scores were used in data analysis.

SPSS 20 for Windows (IBM Corporation, Armonk, NY, USA) was used for statistical analysis. The Kendall coefficient of concordance (*W*) with 95% confidence interval (CI) was generated to estimate reliability. This coefficient is appropriate for assessing strength of agreement where ordinal data and more than two raters are involved (Gisev, Bell, & Chen, 2013). Reliability coefficients were interpreted using the Landis and Koch standards for kappa values (Landis & Koch, 1977) that had been extended to *W* values: 0.81–1.00, almost perfect; 0.61–0.80, substantial; 0.41–0.60, moderate; 0.21–0.40, fair; 0.00–0.20, slight (Gisev et al., 2013). Because the UMCT produced ordinal‐level data and FTSST scores were not normally distributed, relationship between UMCT‐KE scores and FTSST scores was determined using the Spearman rho (ρ) (Portney & Watkins, 2009). Correlation coefficients were interpreted using the cutoff suggested by Terwee et al.: ≥0.50, positive; <0.50, negative (Terwee et al., 2007).

## 3 RESULTS

Twenty‐nine adults (mean age* *=* *55 ± 8 years; men = 72%, 21/29) with chronic stroke (mean time since stroke onset* *=* *9 ± 5 years, range* *=* *1–21 years) participated. Fifty‐five percent (16/29) had left hemisphere stroke or right‐sided muscle weakness. Most of the participants (66%, 19/29) did not require an assistive device or physical assistance from another person while walking. Demographic characteristics of the participants are further detailed in Table 1. All participant data were complete.

**Table 1:** Characteristics of study participants (*n* = 29)

Characteristic	
Age (y), mean (*SD*)	55 (8)
Sex (males), *n* (%)	21 (72%)
Height (m), mean (*SD*)	1.61 (0.09)
Weight (kg), mean (*SD*)	65.49 (9.59)
BMI (kg/m^2^), mean (*SD*)	25.25 (3.55)
Time since stroke onset (y), mean (*SD*, range)	9 (5, 1‐21)
Right‐sided muscle weakness, *n* (%)	16 (55%)
Did not require assistive device or physical assistance for walking, *n* (%)	19 (66%)
FTSST score (s), mean (*SD*)	18.69 (5.72)
Presence of lower extremity muscle spasticity, *n* (%)	
Knee extensors only	7 (24%)
Knee flexors only	8 (28%)
Both knee extensors and flexors	14 (48%)
Grade of muscle spasticity, MMAS score	
Knee extensors, median (IQR)	1 (1)
Knee flexors, median (IQR)	1 (1)

BMI, body mass index; MMAS, Modified Modified Ashworth Scale; FTSST, Five Times Sit to Stand Test.

On two testing sessions, interrater reliability was substantial for both the UMCT‐KE (*W *=* *0.63–0.67, 95% CI 0.42–0.83) and UMCT‐KF (*W *=* *0.72–0.75, 95%CI 0.55–0.88). Across the three raters, the UMCT‐KE demonstrated substantial to almost perfect test‐retest reliability (*W *=* *0.75–0.82, 95% CI 0.55–0.92), while the UMCT‐KF showed almost perfect test‐retest reliability (*W *=* *0.85–0.87, 95% CI 0.72–0.94). Scores on the UMCT‐KE demonstrated a positive inverse correlation with scores on the FTSST (ρ  =  −0.52; *p *=* *.003) (Table 2).

**Table 2:** Interrater and test‐retest reliability of UMCT‐KE and UMCT‐KF, and correlation of UMCT‐KE with FTSST in adults with chronic stroke

	Correlation with FTSST, Spearman ρ	Interrater reliability across three raters, Kendall *W* (95% CI)	Test‐retest reliability for each rater, Kendall *W* (95% CI)
UMCT‐KE			
T1		0.67 (0.48–0.83)	
T2	−0.52^a^	0.63 (0.42–0.81)	
Rater 1			0.82 (0.67–0.92)
Rater 2			0.77 (0.58–0.90)
Rater 3			0.75 (0.55–0.89)
UMCT‐KF			
T1		0.72 (0.55–0.86)	
T2		0.75 (0.60–0.88)	
Rater 1			0.87 (0.75–0.94)
Rater 2			0.85 (0.72–0.93)
Rater 3			0.85 (0.72–0.93)

FTSST, Five Times Sit to Stand Test; T1, first testing session; T2, second testing session; UMCT‐KE, Upright Motor Control Test Knee Extension subtest; UMCT‐KF, Upright Motor Control Test Knee Flexion subtest.

a Statistically significant *p *<* *.05.

## 4 DISCUSSION

Participants in this study were similar to those described in studies with large samples of individuals with chronic stroke (Rodrigues‐Baroni et al., 2014). This study aimed to investigate the reliability of the UMCT‐KE and UMCT‐KF administration on adults with chronic stroke who had lower extremity muscle spasticity, and explore the relationship of the UMCT‐KE with a clinical measure of muscle function. On the basis of the study findings, both the UMCT‐KE and UMCT‐KF have at least substantial relative reliability, and the UMCT‐KE and FTSST assess related constructs or body functions.

The absence of any published study on reliability (Gorgon & Lazaro, 2016) limited direct interpretation of the findings in relation to earlier literature. Interrater agreement and test‐retest agreement for all raters were at least substantial, supporting the stability of UMCT‐KE and UMCT‐KF ratings. Use of a well‐defined testing protocol and standardized instructions, and sufficient rater training are known to contribute to dependable results in reliability studies (Mong et al., 2010; Tuijin et al., 2012). However, wide confidence intervals with most of the lower limits extending down to moderate levels imply that further enhancements in test procedures and/or rater training may be needed. This finding is important because misclassifications can negatively affect the usefulness of the UMCT‐KE and UMCT‐KF in predicting poststroke walking status. The scoring aspects requiring subjective judgment that may introduce unnecessary variations include: (1) gauging whether or not weight‐bearing of the patient on the rater's arms is excessive especially when administering the UMCT‐KE; (2) estimating that the amount of knee flexion has exceeded 60 degrees when administering the UMCT‐KF. Providing additional qualifiers in the UMCT ordinal scale to facilitate scoring and increasing familiarity of raters through longer practice on test administration may further enhance reliability.

The positive direct relationship between scores on the UMCT‐KE, a test of voluntary control of movement, and FTSST, a test of muscle power, suggests that these tests assess related constructs (movement function and muscle function, respectively). Both constructs are classified under the same Body Functions domain (Neuromusculoskeletal and Movement‐related Functions) of the ICF (World Health Organization, 2001). The UMCT‐KE may successfully capture the ability of patients to accept load on the more affected lower extremity, which is an important dimension of sit‐to‐stand performance (Lomaglio & Eng, 2005). The positive relationship between the UMCT‐KE and FTSST builds on evidence from the study by Mercer et al. that showed direct correlations between UMCT‐KE scores and force platform measurements of limb loading (Mercer et al., 2009). This finding warrants more research that will establish the validity of the UMCT‐KE by testing it against other tests and measures under the same and related categories in the ICF framework.

Major limitations of this study were its small sample size and use of convenience sampling. Although the findings were positive overall, use of a larger sample size could have resulted in narrow confidence intervals and therefore better reliability estimates. Due to the use of convenience sampling, a large proportion of the sample comprised higher‐functioning adults with chronic stroke. This feature of the study could limit the generalizability of the results. Lastly, the extent to which the participants mobilized inside the home and in the community was not documented in this study. Higher‐functioning patients likely mobilize more in the upright position and thus may demonstrate more variable UMCT scores. This hypothesis needs to be confirmed in future studies by stratifying the sample based on mobility status (i.e. community ambulators versus household ambulators).

In conclusion, scores on the UMCT‐KE and UMCT‐KF show substantial to almost perfect reliability in adults with chronic stroke, with scores on the UMCT‐KE showing a positive relationship with scores on the FTSST. The relationship of UMCT‐KE scores with FTSST scores implies that the UMCT‐KE can provide information that relates with the construct of muscle function in a weight‐bearing position. The feasibility of test administration in patients with lower extremity muscle spasticity, as shown in this study, enhances the clinical relevance of both subtests.

## CONFLICT OF INTEREST

The authors declare that there is no conflict of interest.
